# Transcriptional signatures of human peripheral blood mononuclear cells can identify the risk of tuberculosis progression from latent infection among individuals with silicosis

**DOI:** 10.1080/22221751.2021.1915184

**Published:** 2021-08-06

**Authors:** Qiao-ling Ruan, Qing-luan Yang, Yi-xin Gao, Jing Wu, Si-ran Lin, Jing-Yu Zhou, Ling-yun Shao, Sen Wang, Qian-qian Liu, Yan Gao, Ning Jiang, Wen-hong Zhang

**Affiliations:** a Department of Infectious Diseases, Huashan Hospital, School of Life Science, Fudan University, Shanghai, People’s Republic of China; b National Clinical Research Center for Aging and Medicine, Huashan Hospital, Fudan University, Shanghai, People’s Republic of China; c Key Laboratory of Medical Molecular Virology (MOE/MOH) and Institutes of Biomedical Sciences, Shanghai Medical College, Fudan University, Shanghai, People’s Republic of China

**Keywords:** Tuberculosis, peripheral blood mononuclear cell, biomarker, latent tuberculosis infection, RNA sequencing, interferon-gamma

## Abstract

Host immune factor plays an important role in the progression of latent tuberculosis infection (LTBI) to active tuberculosis (TB) disease. However, whether global gene expression measured in blood biomarkers allows the identification of prospective signatures for TB risk remains unknown. Hence, we aimed to assess the ability of the transcriptome signatures in the human peripheral blood mononuclear cells (PBMCs) of LTBI subjects to differentiate future TB progressors from non-progressors. In a randomized clinical trial of TB preventive treatment of 513 participants with silicosis, we randomly collected PBMC samples from 50 LTBI subjects in the observational group, which was monitored for TB disease progression for 37 months. The prospective signatures of TB risk between the two participants who developed active TB (progressors) and four matched individuals who remained healthy (non-progressors) were compared using differential expression analysis, Gene Ontology analysis, Kyoto Encyclopedia of Genes and Genomes pathway analysis, and Weighted Gene Co-expression Network Analysis. The 20 TB-specific differentially expressed genes, which were significantly downregulated in TB progressors, were revealed to be associated with interferon-gamma response-related genes. Therefore, the PBMC transcriptome profiles analyzed in this study may help identify LTBI individuals who are at risk of progressing to active TB among silicosis patients and may provide new insights for targeted intervention to prevent disease progression.

**Clinical trial registration:**

www.clinicaltrials.gov (NCT02430259)

## Introduction

Tuberculosis (TB) remains as one of the most important infectious diseases worldwide, with an estimated global burden of ∼1.7 billion (23.0%) for latent tuberculosis infection (LTBI) [1]. The lifetime risk of developing active TB for a documented LTBI person is estimated to be only 5%–10%. Exposure to silica dust or the development of silicosis predisposes to *Mycobacteria tuberculosis* infection, and the TB risk increases with the severity of silicosis [2]. Currently, diagnosis of LTBI involves assessment of the reactivity to mycobacterial antigens, as determined by a tuberculin skin test (TST) or an *M. tuberculosis* (MTB)-specific interferon-gamma (IFN-γ) release assay (IGRA), which can demonstrate the elicitation of a T cell-mediated immune response caused by the infection. IGRA is more reliable and has higher specificity due to low cross-reactivity with most non-tuberculous mycobacteria and in cases with a previous history of vaccination with the Bacille Calmette-Guérin vaccine [3]. However, these tests cannot determine whether the infection has been cleared, whether the infection is controlled in an individual, whether the patient might have a subclinical disease, or whether the patient will develop active TB; therefore, neither IGRA nor TST can accurately predict the risk of active TB development [4–7]. Furthermore, these methods cannot completely describe the spectrum of infectious states after MTB exposure.

Previous studies have revealed that the heterogeneity of latent tuberculosis can be defined by the blood transcriptome signature, which may provide information on the temporal changes in host immunity that are associated with active TB disease progression. Earlier identification of LTBI patients with high risk of TB progression has considerable potential for targeted preventive therapy and may provide novel strategies to curb the transmission of MTB. Several sets of blood transcriptomic signatures associated with TB risk have successfully identified LTBI subjects who progressed to active disease ≤18 months before TB diagnosis [8,9]. However, consensus has not been achieved regarding the optimal reduced gene sets as potential diagnostic biomarkers for the accurate identification of people at risk of developing TB before the onset of symptoms.

In the present study, we used RNA sequencing (RNA-seq) to assess the transcriptional signature of peripheral blood mononuclear cells (PBMCs) and to determine its potential in identification of the subgroup at a risk of progression to active TB among the LTBI subjects with silicosis. Better understanding of the different immune responses to LTBI in people with controlled infection (non-progressors) and those who develop the disease (progressors) may help in the prevention of TB.

## Material and methods

### Study cohort and design

**Figure 1.**
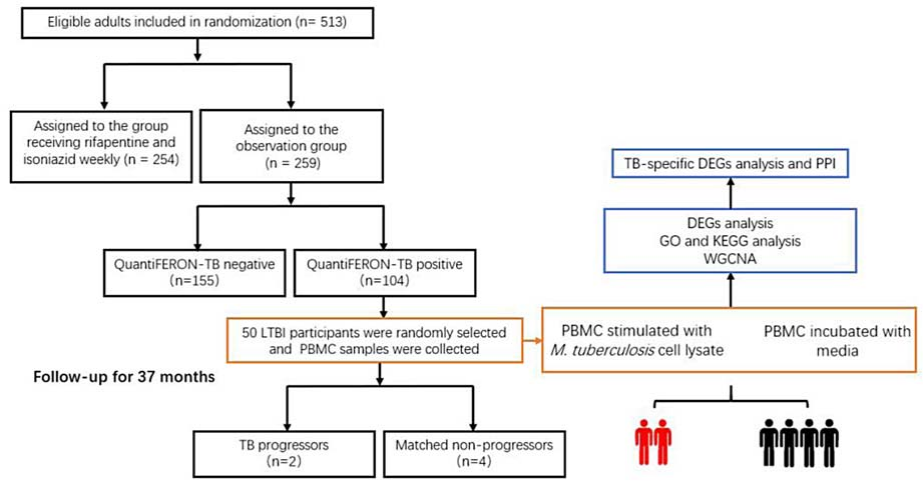
Flow diagram of data collection and analysis. PBMC: peripheral blood cells; GO analysis: Gene Ontology analysis; KEGG analysis: Kyoto Encyclopedia of Genes and Genomes analysis; WGCNA: Weighted correlation network analysis; GSEA: Gene-set enrichment analysis.

This is a sub-study of an open-label, randomized clinical trial which evaluated the efficacy and tolerability of weekly rifapentine and isoniazid medication for three months to prevent TB in individuals with silicosis (ClinicalTrials.gov number: NCT02430259) [10]. In the study, from February to April 2015, 513 patients aged 18–65 years were randomly assigned between the preventive treatment (*n *= 254) and observation groups (*n *= 259). Twenty-eight participants were diagnosed with active TB, 9 and 19 in the preventive treatment group and observation groups after 37 months follow-up. Patients with immunosuppression, with a previous history of TB or treatment for LTBI, and those with HIV were excluded from the present study. LTBI was screened using QuantiFERON Gold-In-Tube (QFT). Fifty participants were randomly selected according to simple unrestricted randomization form 104 participants with LTBI of the observation group. After obtaining informed consent, these 50 individuals were prospectively enrolled and sampled, monitored for 37 months after enrolment, examined every six months, and screened annually for active TB. Active TB during longitudinal assessment was diagnosed based on microbiological confirmation of MTB by culture or positive Xpert MTB/RIF (Cepheid, Sunnyvale, CA, USA). Two LTBI subjects who developed active TB (progressors) and four LTBI subjects who did not develop active TB (non-progressors) were enrolled after adjusting for sex, age, silicosis stage, and comorbidities (Table 1 and Figure 1).

**Table 1.** Demographic and clinical features of two TB progressors and four non-progressors.

No. patient	Sex	Age	Silicosis categories^a^	BMI	Current smoker	Comorbidities	Long-term drug use (including steroid)	TB diagnosis	Time to TB/follow-up time (months)
107	Male	65	3	22.95	Yes	None	None	Culture	27
129	Male	55	3	24.69	No	None	None	GeneXpert.TB	36
162	Male	58	3	22.15	No	None	None	–	37
165	Male	54	3	21.93	Yes	None	None	–	37
170	Male	50	3	25.08	Yes	None	None	–	37
173	Male	64	3	24.62	No	Gallbladder stone	None	–	26

^a^ Silicosis categories were determined according to the revised edition (2011) of ILO Guidelines for the use of the ILO International Classification of Radiographs of Pneumoconioses.

The study protocol was approved by the ethics committees of the First People’s Hospital of Wenling, Zhejiang, China. All participants in the parent trial agreed to participate in this study and provided written informed consent.

### PBMC preparation and antigenic stimulation

Human PBMCs were purified, collected, and diluted to 2.5 × 10^6^ mL^−1^ using AIM-V. The PBMCs (500 μL/well) were incubated with 10 ng/mL MTB (*H37Rv*) cell lysate in culture media at 37°C with 5% CO_2_ for 20–24 h. The synchronous PBMCs were incubated without antigen in culture media as un-stimulated samples. The culture supernatant was discarded, and the cells at the bottom of the well were resuspended with 1 mL Trizol. The cells were harvested for subsequent RNA extraction experiments.

### Library preparation and RNA-seq

Total RNA was extracted from peripheral blood samples using the RNA Easy Mini Kit (Qiagen, Hilden, Germany) following the manufacturer’s instructions. The RNA integrity number (RIN) was measured using the Agilent Bioanalyzer 2100 system (Agilent Technologies, Santa Clara, CA, USA). Only samples with RIN values >8 were used to prepare the RNA-seq library. Ribosomal RNA (rRNA) was removed using the Ribo-Zero rRNA Removal Kit (Epicentre, Madison, WI, USA). The strand-specific sequencing libraries were constructed using the NEBNext® Ultra^™^ II Directional RNA Library Prep Kit for Illumina® (New England Biolab, Inc., Ipswich, MA, USA), according to the manufacturer’s instructions. The lncRNA-seq libraries were sequenced (paired-end, PE150) using the Illumina HiSeq X Ten platform (Illumina, San Diego, CA, USA), with ∼40 million reads obtained for each sample.

The gene expression patterns in 12 PBMC samples derived from the 6 participants using RNA-seq. Two PBMC samples were obtained from each participant, one as the control (unstimulated) and one stimulated with the MTB cell lysate.

### RNA-seq data pre-processing, differential expression analysis, and functional enrichment analysis

The quality of the RNA-seq data obtained was assessed using FastQC [11]. The raw sequencing reads were pre-processed by trimming the adapter sequences and by removing the >20 base pair (bp) long low-quality reads (Phred quality score <20). The filtered clean reads were aligned to the human reference genome (GRCh38) using Tophat2 [12]. Then, uniquely mapped reads were assigned to each annotated gene using featureCounts [13]. Differential expression and statistical analyses were performed using DESeq2 from the R package [14]. Annotated sequences with absolute log2-transformed fold changes (log2FC) >1 and *P*-values <0.05 were considered as differentially expressed genes (DEGs). Principal component analysis was performed on all normalized expression data using prcomp package with R. The PCA plot demonstrated top 2 PCs for each sample.

To determine the Gene Ontology (GO) terms [15] and Kyoto Encyclopedia of Genes and Genomes (KEGG) pathways [16] associated with the DEGs, functional enrichment analysis (PCA) was conducted using the clusterProfiler package [17], with the threshold set to *P*-value <0.05. GO terms were used to describe gene functions and to classify the DEGs into three functional categories, namely biological process (BP), cellular component (CC), and molecular function (MF).

### Weighted correlation network analysis (WGCNA)

A co-expression network was constructed (*β*-value = 18), in which genes were clustered into branches of highly expressed genes and modules were identified by the tree cut algorithm with the additional PAM stage. Two binary variables, TB progressor and non-progressor, were generated and used to calculate the module trait relationships in both MTB lysate unstimulated and stimulated samples. Additionally, a between-group Kruskal–Wallis test was applied to the Module Eigengene (ME) values to detect overall differential gene expression. The modules were then functionally annotated using clusterProfiler package.

### Protein–protein interaction (PPI) network construction

The PPI data were obtained from the Search Tool for the Retrieval of Interacting Genes (STRING) database. The PPI network was constructed using the TB-specific DEGs, with a confidence score of >0.9. The gene networks were subsequently generated using STRING: functional protein association networks v11 [18] to further assess the complex associations among the TB-specific DEGs.

## Results

### Different gene expression patterns between TB progressors and non-progressors

**Figure 2.**
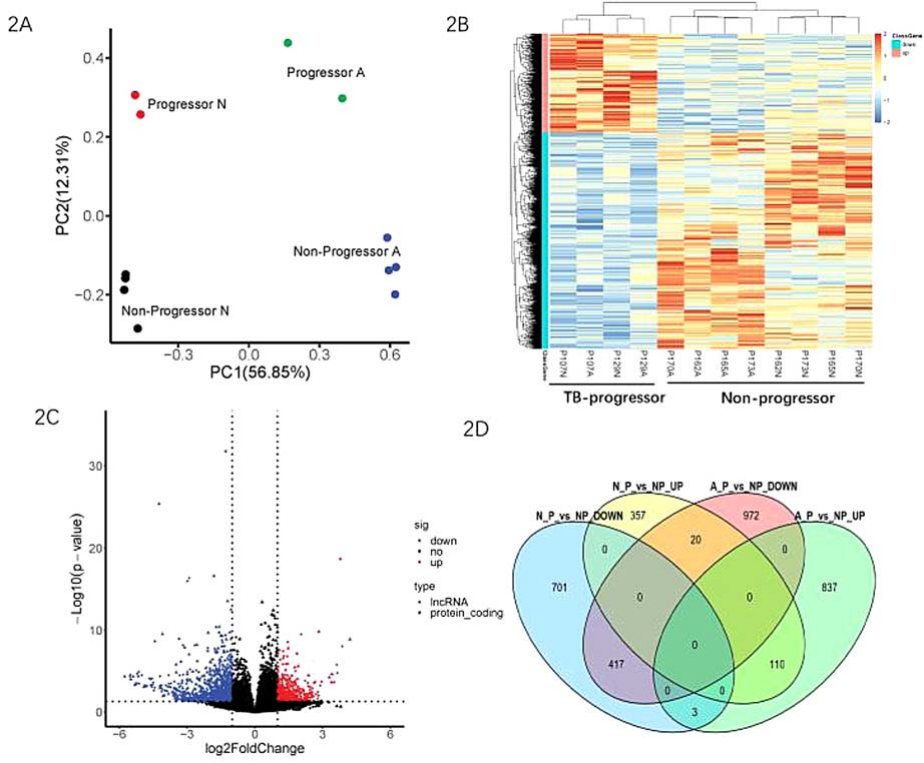
Transcriptional patterns of peripheral blood mononuclear cells defining stimulated and un-stimulated, TB progressors and non-progressors. (A) Principal component analysis diagram. (B) Unsupervised hierarchical clustering of transcribed genes and differentially expressed over 1.5-fold for 12 PBMC samples. (C) Volcano plot shows the upregulated and downregulated transcribed genes between TB progressors and non-progressors. (D) Venn diagrams showing overlaps of TB-specific genes changes between TB progressors and non-progressors. TB progressors: P107 and P129; Non-progressors: P102, P105, P170 and P173. A: PBMCs stimulated by TB antigen, N: PBMCs incubated without TB antigen.

RNA-seq of the messenger RNAs (mRNAs) and lncRNAs provided data on a total of 16,015 mRNAs and 15,787 lncRNAs. The PCA plot based on gene expression profiles clearly distinguished between the stimulated and unstimulated samples, as well as the TB progressors and non-progressors (Figure 2(A)). A total of 1608 DEGs were identified in the unstimulated samples, with 487 upregulated and 1121 downregulated DEGs in TB progressors. On the other hand, 2359 DEGs were identified in the stimulated samples, with 950 upregulated and 1409 downregulated DEGs in TB progressors. Heatmap analysis by hierarchical clustering illustrated the gene expression profiles between the stimulated and unstimulated samples and between TB progressors and non-progressors (Figure 2(B)). The expression profile of TB progressors was highly distinct from the non-progressors. The distribution of all DEGs was plotted on a volcano map using -log10 false discovery rate (-log10FDR) and log2FC values (Figure 2(C)). Furthermore, the DEGs that were common in TB progressors and non-progressors and in stimulated and unstimulated samples were identified (Figure 2(D)), which revealed that most DEGs were unique to a single pairwise comparison. In particular, 972 downregulated and 837 upregulated TB-specific DEGs, which were differentially expressed in the stimulated samples only, were also identified in TB progressors.

### Functional characterization of DEGs

**Figure 3.**
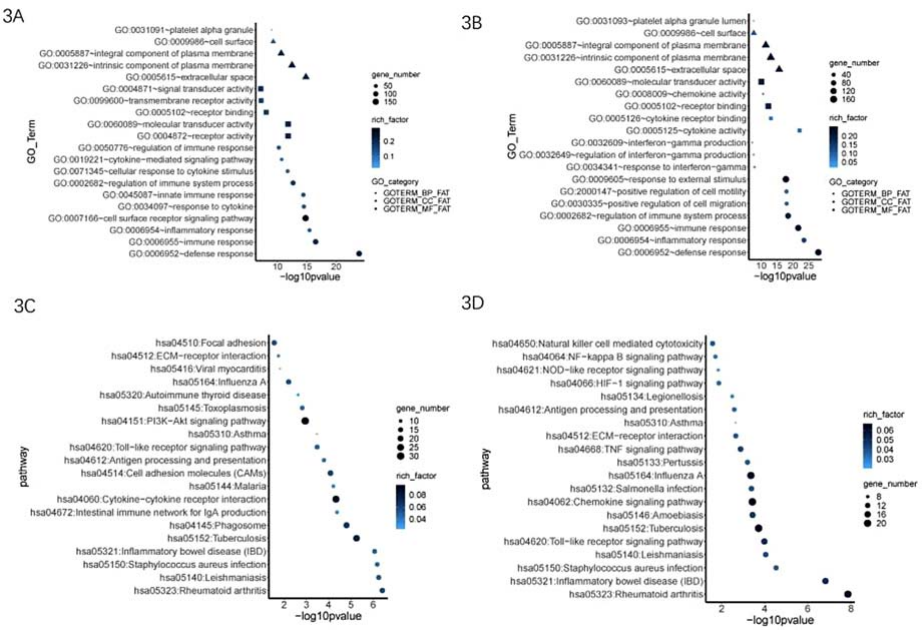
Different gene expression and functional enrichment analysis of TB progressors and non-progressors using un-stimulated (left) and stimulated (right) samples. (A–B) DEGs was applied to GO analysis in BP, CC and MF. (C–D) DEGs was applied to KEGG analysis.

To characterize the biological functions of the DEGs between the TB progressors and non-progressors, GO analysis was performed. The top enriched GO terms in BP comprised processes predominately related to the immune response (Figure 3(A) and (B)). Most DEGs involved in the immune response were downregulated in TB progressors, thereby indicating the absence of host defense against MTB in these individuals. Interestingly, DEGs related to IFN-γ were only enriched in the stimulated samples. The five most enriched GO terms in CC and MF were also identified (Figure 3(A) and (B)). Additionally, KEGG pathway analysis revealed that most DEGs in both unstimulated and stimulated samples were associated with various pathways, including the pathway for tuberculosis (Figure 3(C) and 3(D)).

**Figure 4.**
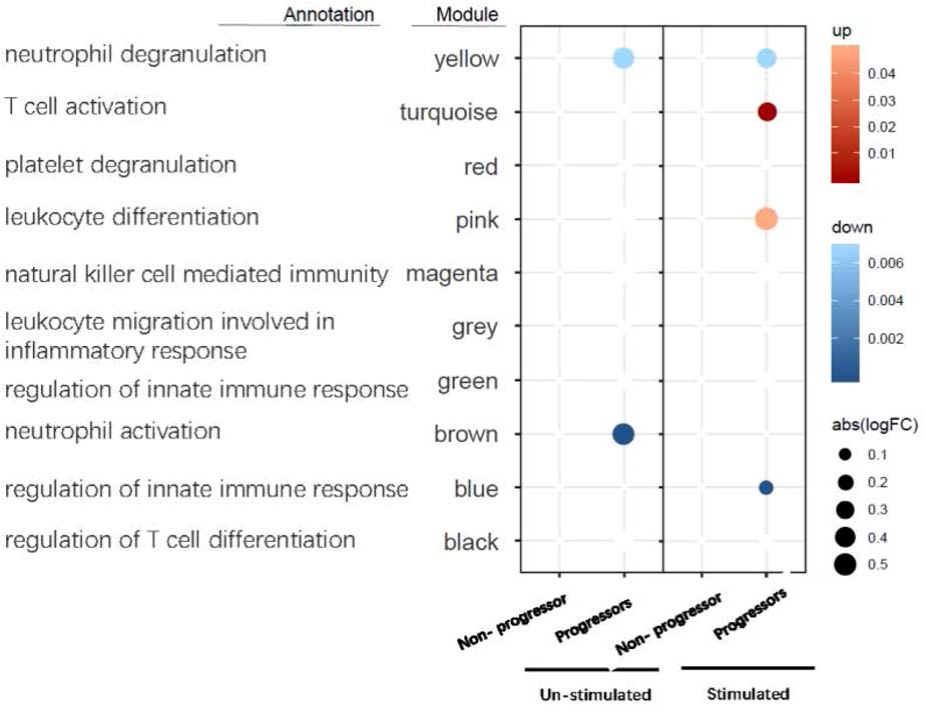
Modular transcriptional signatures of TB progressors compared to non-progressors. Fold enrichment scores derived using QuSAGE are depicted, with red and blue indicating modules over- or under-expressed. Colour intensity and size represent the degree of enrichment.

To assess the gene expression profiles associated with TB progression, WGCNA was performed. The genes were clustered according to their co-expression, thereby revealing a network with 10 co-expression modules (Supplementary Fig S1). The ME value, which is the first principle component that functions as a representative of the module, was calculated from each module. The ME values were correlated with the variables that represented the TB progressors and non-progressors. Results showed that the ME values of the turquoise and pink modules were positively correlated with the TB progressors in the stimulated samples, while the ME value of the blue module was negatively correlated with the TB progressors in the stimulated samples. The top enriched GO terms associated with the turquoise and pink modules were “T cell activation” and “leukocytes differentiation,” while the top GO term associated with the blue module was “regulation of innate immune response” (Figure 4). Overall, these results suggested that T cell activation genes were more highly expressed than innate immune response genes in TB progressors.

### PPI networks in TB-specific DEGs

**Figure 5.**
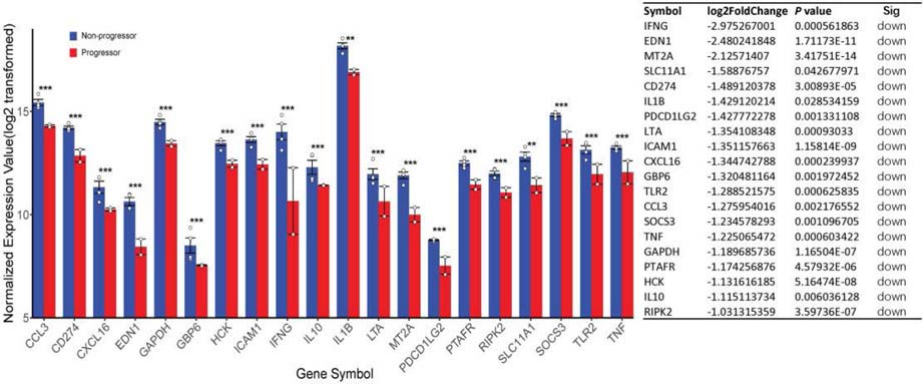
Normalized expression value of twenty discriminatively expressed TB-specific genes related with type II interferon between TB progressors (red) and non-progressors (blue). Kruskal–Wallis tests were used to compare the differences among the two groups. **Significant difference: 0.001 < *P* < 0.01; ***Significant difference: *P* < 0.0001. Log2 fold changes and *P* value were listed on the right.

Based on the findings of DEGs and their enriched functions, innate immune response in stimulated samples were heighted in TB-progressors. To narrow down the related DEGs in TB progression, STRING was used to construct the PPI network and to explore the associations of genes (at the protein level) involved in TB progression among LTBI individuals. The TB-specific DEGs between TB progressors and non-progressors were only differentially expressed in the stimulated samples, which were more relevant to the host immune response to MTB*.* A total of 972 downregulated and 837 upregulated TB-specific DEGs comprised two sub-networks. Sub-network1 was larger, with 186 nodes and 726 edges (Supplementary Fig S2), while sub-network2 had 11 nodes and 78 edges. Among the DEGs identified, we found that the genes associated with the IFN-γ pathway, including *IFNG, EDN1, MT2A, SLC11A1, CD274, IL1B, PDCD1LG2, LTA, ICAM1, CXCL16, GBP6, TLR2, CCL3, SOCS3, TNF, GAPDH, PTAFR, HCK, IL10,* and *RIPK2*, were highly enriched. Interestingly, these genes were significantly downregulated in TB progressors (Figure 5), suggesting the absence of IFN-γ response to MTB after TB-specific antigenic stimulation.

## Discussion

The heterogeneity of LTBI was suggested in previous studies, which indicated a heterogeneous risk of developing TB. In the present study, we confirmed that the transcriptional signatures of latent TB-infected participants with different TB risks were significantly different. We found that there were different gene expression profiles between TB progressors and non-progressors in both the stimulated and unstimulated PBMC samples of participants with LTBI. The DEGs were also found to be functionally associated with immune, defense, and cellular responses and several KEGG pathways, including the pathway for TB. PPI network construction further revealed that TB-specific DEGs were associated with IFN-γ-related pathways, in which all 20 TB-specific DEGs were significantly downregulated in TB progressors. These results suggest that it is possible to predict the progression from latent to active TB disease using PBMC baseline gene expression.

The infection of MTB can result in three different outcomes – active disease, latent infection, and clearance – depending on both pathogen and host immune responses. However, the immune response mechanism elicited in the host after MTB exposure is not fully elucidated, especially since there is limited information about host factors that determine infection control versus progression. Blood transcriptomic profiling may provide an unbiased analysis and comprehensive overview of the host factors that are related to tuberculosis status after MTB infection. RNA-seq is a transcriptome-based technology that is quantitative, sensitive, and unbiased. A longitudinal transcriptomics analysis of cynomolgus macaques infected with MTB, which represent the spectrum of clinical outcomes observed in human TB patients, reported an increased transcriptional activity of genes encoding molecules involved in innate and adaptive immunity [19,20]. Hence, it appears that the transcriptional profiles of immune response might influence the fate of infection.

In previous studies, the predictive signatures were usually obtained through the transcriptomic analysis of peripheral whole blood, which can be sampled with convenience. In a longitudinal analysis of South African adolescents with LTBI, Zak et al. [8] identified a 16-gene expression signature for prediction of the risk of TB disease progression from whole blood. In another study, Suliman et al. [9] identified and validated a four-gene signature, RISK4 (*GAS6*, *SEPT4*, *CD1C*, and *BLK*), to predict the risk of progression to active TB disease in diverse African cohorts recently exposed to TB (up to 2 years before disease onset). Singhania et al. [21] found that a whole blood TB-specific 20-gene signature was only minimally enriched in most IGRA- contacts and transiently enriched in the IGRA+ group, which did not progress to active TB. Among the gene signatures previously validated, only two genes, *SEPT4* and *SCARF1*, were common. Thus, the transcriptomic profile from whole blood may not accurately represent the pathogenic events in the lungs or the specific changes in immune response during MTB infection. To minimize unrelated background noise and to simultaneously maximize MTB*-*specific host immune response, we analyzed the MTB lysate-stimulated and unstimulated PBMC samples collected from LTBI subjects. RNA-seq revealed a significant difference in gene expression patterns between the stimulated and unstimulated samples, thereby indicating that the stimulation using MTB lysate was effective. The differential gene expression pattern in TB progressors was also found to be distinct from non-progressors, suggesting that the transcriptomic pattern was different in individuals with latent infection with different TB risks. As expected, genes involved in innate and acquired immune responses were differentially expressed. Additionally, KEGG pathway analysis revealed that the pathway for tuberculosis was enriched in both the stimulated and unstimulated samples.

The IFN-inducible signature is not common to all inflammatory responses but is preferentially induced in some diseases, which may potentially reflect host protection or pathogenesis. In a previous study, a transcriptomic signature dominated by IFN-inducible genes was identified in the whole blood of patients with active TB, but not in healthy controls or subjects with LTBI [22]. This IFN-inducible gene signature include genes downstream of both type I and type II interferons. High and sustained levels of type I IFNs (IFN-αβ) from the macrophage infected with MTB have a deleterious effect in the control of TB [23]. On the other hand, type II IFNs (IFN-γ) has protective immune responses to MTB bacilli [24]. After exposure to MTB-specific antigen, the attenuated host type II IFN response may result in disease progression. Among the IFN-γ response-related genes, *IFNG* (which encodes IFN-γ) was found to be significantly downregulated in future TB progressors. When MTB invades the host, the innate immune response is activated, which includes the production of IFN-γ by natural killer (NK) and NK T cells. IFN-γ subsequently activates the macrophages, which act as the first line of host defense against the pathogen [25]. Once antigen-specific immunity develops, IFNG is produced by CD4 and CD8 T cells [26]. Previous reports have indicated that mice with disrupted IFNG production are more likely to be infected with MTB compared with wild-type mice [27]. The critical role of IFNG in controlling MTB infection was also previously reported in humans [28]. Thus, LTBI individuals who suffer from inherited disorders of IFNG-mediated immunity after TB-specific antigen stimulation are more likely to develop active TB. In this study, another IFN-γ response-related gene, *EDN1*, was also significantly downregulated in TB progressors. EDN1 is a well-known vascular regulator; its specific role in infectious diseases, including tuberculosis, are currently being elucidated. A mice model showed that the inhibition of *EDN1* activity by antagonism during MTB infection resulted in an increased number and a greater severity of lung lesions, as well as an increased bacterial burden [29]. Furthermore, *EDN1* is one of the macrophage host transcriptional enhancers during MTB infection that drives macrophage response via transcriptional activation of key immune genes, such as *TNF* and *CCL3* [30], which are also significantly downregulated in TB progressors. *SLC11A1*, a crucial determinant susceptible gene of TB in mice and the most widely studied candidate gene for TB susceptibility in non-HLA genes, was also revealed to be significantly downregulated (log2FC = 1.5) [31]. Other genes involved in IFN-γ response, such as *CD274*, *IL1B, PDCD1LG2,* and *ICAM1*, were also proven to play a role in innate immunity against TB [32–34]. Therefore, our results suggested that LTBI individuals with insufficient type II IFN response after exposure to MTB were more likely to develop active TB.

Despite the new insights provided by our study, there are some limitations that must be addressed. First, the baseline PBMC samples of LTBI subjects who further developed active TB are difficult to collect because of the low incidence rate of active TB among those subjects. So only two cases TB progressors were included for transcriptome for analysis, which might have resulted in reduced statistical power. However, the two TB progressors and four controls were well matched after adjustment for age, sex, HIV coinfection, and previous TB disease. Further, in the process of screening DEGs, genes with low expression levels in all samples were filtered out and we not only considered the *P* value, but also the log2FC differences between sample groups. In addition, the PCA plot, hierarchical clustering (heatmaps), and Venn diagrams showed that the transcriptomic signatures were significantly different in different groups. Thus, we think that the transcriptome data were reliable for analysis. Second, the analysis only focused on the subgroup with IGRA-defined LTBI, despite a proportion of prospective TB cases developing in subjects with IGRA-negative at baseline. However, investigation of the spectrum of LTBI using transcriptome analysis can accurately discover the underlying host immune mechanisms involved; it is also practical to further assess the risk of TB development among individuals with LTBI under clinical settings. Third, we did not perform PCR-based validation for an accurate representation of the transcriptome profiles in TB progressors and non-progressors. All these enrolled subjects were at stage 3. Thus, we can speculate that the silicosis status did not contribute to the observed varied transcription profiles between the two groups. But more studies are needed to investigate whether the transcription profiles are different in patients with different silicosis categories. The finding of the present study should be interpreted with caution in on the population. In future, similar studies are warranted in other TB risk population besides silicosis patients. Researches on the mechanism of how impaired IFN-γ-pathway lead to tuberculosis progression are necessary.

The present study provides new evidence that individuals with LTBI who progress to active TB (progressors) exhibit an immune response which is different from individuals with controlled infection (non-progressors). Understanding the immunological heterogeneity by studying the transcriptome profiles in PBMCs may help elucidate the nature of protective immunity and may provide new insights for facilitation of targeted preventative therapy for people at a high risk of developing TB.

## Supplementary Material

Sup_2.tif

Sup_1.tif

## Data Availability

The data that support the findings of this study are available from the corresponding author upon reasonable request.
