# Supplementary figures and images for: Transcriptional signatures of human peripheral blood mononuclear cells can identify the risk of tuberculosis progression from latent infection among individuals with silicosis
Source: Emerg Microbes Infect. 2021 Aug 6;10(1):1536–44. doi: 10.1080/22221751.2021.1915184 (PMC8354161; doi:10.1080/22221751.2021.1915184)

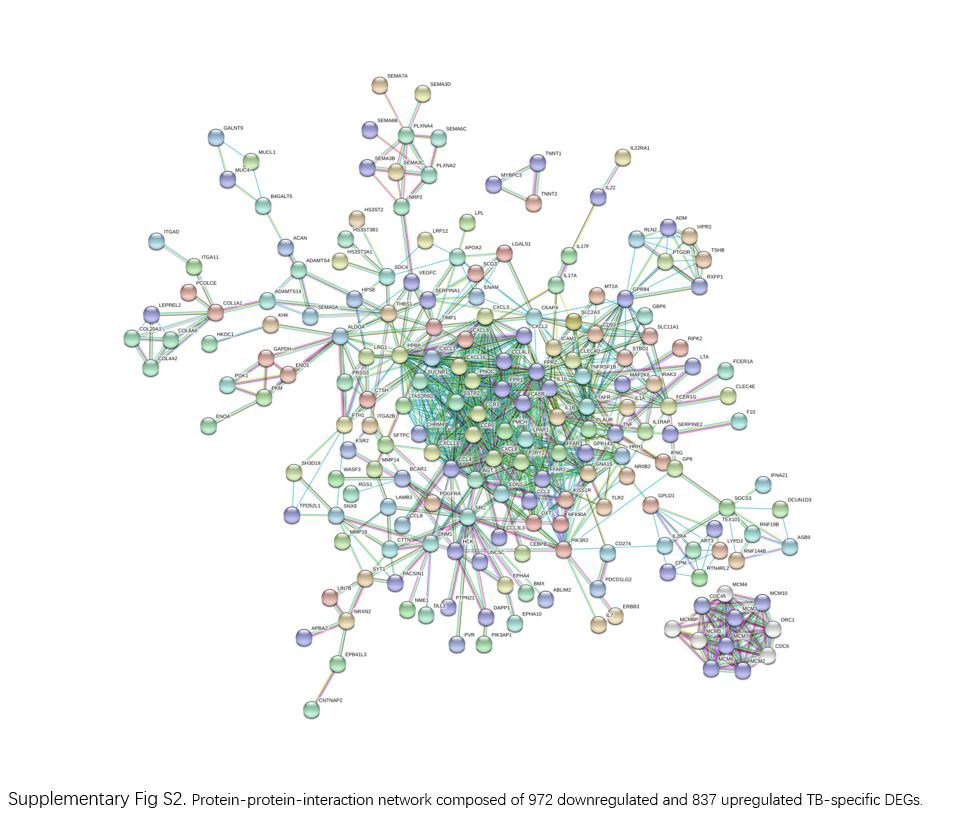

Supplement: Sup_2.tif [file TEMI_A_1915184_SM2830.tif]

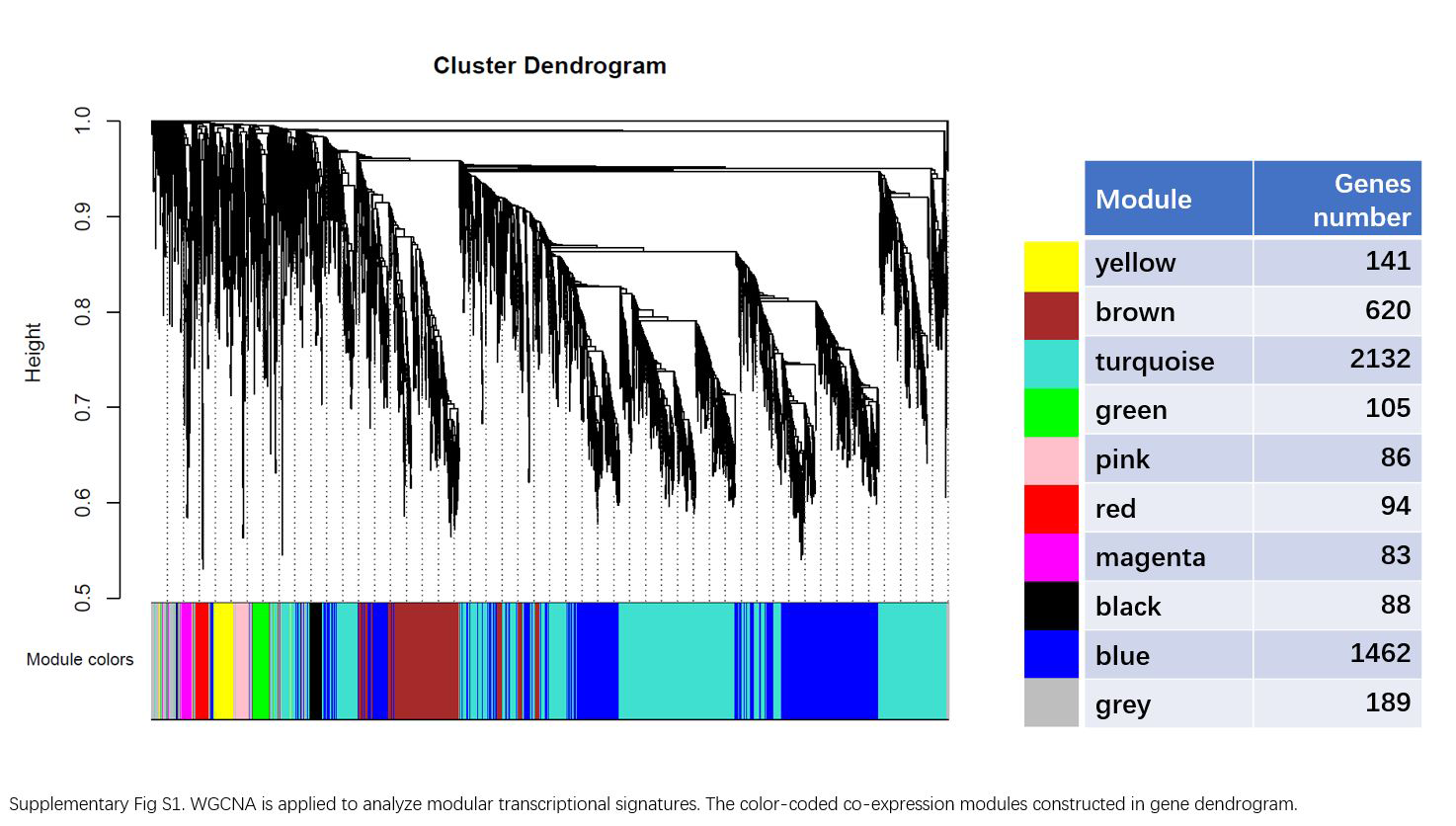

Supplement: Sup_1.tif [file TEMI_A_1915184_SM2829.tif]
